# Dynamics of expenditure on insecticides in the management and control of corn leafhoppers (*Dalbulus maidis*)

**DOI:** 10.1002/ps.70058

**Published:** 2025-07-15

**Authors:** Esther Oliveira Moritz, Wander Plassa, Ivan Bordin, Rodolfo Bianco, Tiago Santos Telles

**Affiliations:** ^1^ Universidade Estadual de Londrina Londrina Brazil; ^2^ Instituto de Desenvolvimento Rural do Paraná – IAPAR‐EMATER Londrina Brazil

**Keywords:** corn, corn stunt complex, agricultural sustainability, economic impact

## Abstract

**BACKGROUND:**

The corn leafhopper (*Dalbulus maidis*) has caused significant losses to corn crops, mainly due to the transmission of pathogens of the corn stunt complex. Therefore, our study aimed to estimate the costs of insecticides for controlling this pest. For this purpose, we used primary data from the Paraná Agribusiness Defense Agency, including information on products applied exclusively in the management of the leafhopper. Our estimates were based on the volume of insecticides applied, multiplied by the respective market price, for each agricultural year and municipality, covering the period from 2015–2016 to 2020–2021. To identify spatial patterns, Exploratory Spatial Data Analysis (ESDA) was performed using a first‐order queen‐type contiguity matrix. The techniques applied included Global Moran's I and Local Indicator of Spatial Association (LISA), allowing the identification of spatial patterns and clusters in the state of Paraná.

**RESULTS:**

Between the agricultural years 2015–2016 and 2018–2019, insecticide expenditures (GCC) in most municipalities in Paraná were up to US$ 10.00 ha^−1^. However, in the agricultural years 2019–2020, 2020–2021, and 2021–2022, GCC increased, exceeding US$ 120.00 ha^−1^ in some municipalities. High‐High clusters were identified in the northwest and central regions of Paraná, with displacement to the south and southeast regions in the final year analyzed.

**CONCLUSION:**

The largest GCC were recorded in small cultivation areas, suggesting limitations in technology and technical assistance. Despite the increase in the number of products used, expenditures remained concentrated in a few chemical compositions throughout the analyzed period. © 2025 The Author(s). *Pest Management Science* published by John Wiley & Sons Ltd on behalf of Society of Chemical Industry.

## Graphical abstract

The results reveal an increase in insecticide expenditures for controlling corn leafhoppers, highlighting the need for integrated pest management and sustainable agricultural practices to mitigate economic and environmental impacts.

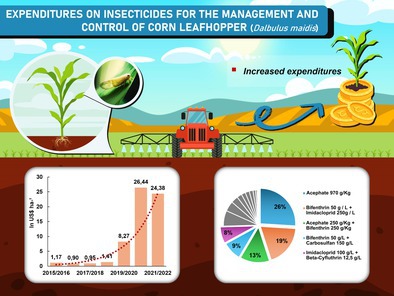

## 1 INTRODUCTION

Corn (*Zea mays*) is the most widely produced cereal in the world. In 2021–2022, global corn production exceeded 1.1 billion tons, with Brazil accounting for 10% of global production.^1^ Corn is a commodity of great economic importance, since its use ranges from human and animal nutrition to the high‐tech industry. Its use in animal feed occurs especially in the poultry, swine, and dairy farming industries,^2^ and it is an essential raw material in the production of ethanol, beverages, and syrups.^3^

In Brazil, during an agricultural year, corn is produced both in the first and second crops. The predominant grain production system in Brazil is the succession cropping system, with soybean in the first crop and corn in the second crop. However, consecutive corn crops create an environment conducive to the continuous reproduction of pests and diseases,^4^ including the corn leafhopper.

The corn leafhopper (*Dalbulus maidis*) has had a negative impact on corn crop productivity.^5^, ^6^ Its impact is mainly due to its ability to transmit pathogens belonging to the corn stunt complex,^7^ such as: (i) red stunting, caused by the phytoplasma *Candidatus Phytoplasma asteris*; (ii) pale stunting, caused by the spiroplasma *kunkelii*; (iii) corn stripe, caused by the virus *Maize Rayado Fino Virus*; and (iv) *Maize striate mosaic virus* (MSMV). Depending on the susceptibility of the cultivars, pathogens of the corn stunt complex can lead to yield losses of up to 70%, especially when infection occurs during the early phase of plant development.^8^

The management of the corn stunt complex occurs mainly through the prevention of infection by pathogens. The main recommended management measures include planting of cultivars tolerant to the stunt complex,^9^ control of volunteer corn and other host grasses to prevent insect multiplication, the formation of sources of pathogen inoculum in the off‐season,^7^ and the control of corn leafhoppers using insecticides, mainly at the beginning of plant development, which is the period in which the plant is most susceptible to insect attack.^10^

However, to date, no effective protocol has been established for controlling the corn leafhopper, and the incidence of the insect has increased in agricultural areas throughout Brazil,^4^ leading producers to intensify the use of insecticides, due to fear of productivity losses. Thus, the hypothesis of this study is that over the years there has been an increase in spending on insecticides to control corn leafhoppers in the state of Paraná, especially in regions where there is greater corn production. On the one hand, productivity losses due to the corn stunt complex can lead to reduced revenue, while, on the other hand, the intensification of insecticide use leads to increased spending on controlling the insect vector.

Although this is a highly relevant topic when considering the sustainability of production, given that the corn leafhopper has high potential for proliferation and dispersion and, consequently, negative impacts on production, in relation to the use of insecticides, studies that make a connection between the incidence and management of this pest in crops and the economic impacts generated by its control are rare. Thus, the objective of the current study was to verify the dynamics of expenditure on insecticides for controlling corn leafhoppers.

## 2 MATERIAL AND METHODS

**Figure 1:**
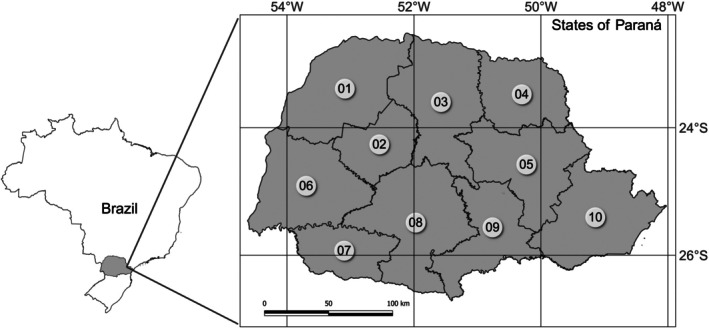
Geographical location of the state of Paraná and its division into mesoregions. 01, northwest Paraná; 02, central‐west Paraná; 03, north central Paraná; 04, Pioneer north Paraná; 05, central‐east Paraná; 06, west Paraná; 07, southwest Paraná; 08, central‐south Paraná; 09, southeast Paraná; 10, Metropolitan of Curitiba.

The study was carried out in the state of Paraná (Fig. 1), using the database of the Monitoring System for Trade and Use of Agrochemicals in the State of Paraná (SIAGRO), from the Paraná Agribusiness Defense Agency (ADAPAR). SIAGRO is a computerized tool that allows professionals who issue agronomic prescriptions and those who sell agricultural products to input information about all pesticides sold in Paraná.

To estimate the costs of insecticides for corn leafhopper (*D. maidis*) control agronomic prescriptions issued, in which the target pest was exclusively the corn leafhopper, in the agricultural years 2015–2016 to 2021–2022, were selected. The data used in this study include: date the product was commercialized, municipality where the product was applied, commercial name of the product (used only for price survey), chemical composition of the product, and quantity commercialized in liters (L) or kilograms (kg).

After organizing the data, seed treatment products were disregarded, since these, even if prescribed to control corn leafhoppers, have a broad spectrum of protection against insects that can cause damage in the initial phase of corn crops, such as caterpillars and bugs. Therefore, the estimate of expenditure on insecticides to control corn leafhoppers was based only on pesticides applied in the field.

To estimate spending on insecticides to control corn leafhoppers, data on prices paid for insecticides by the National Supply Company (CONAB), the Secretariat of Agriculture and Supply of the State of Paraná (SEAB),^11^ and companies in Paraná specializing in the sale of agricultural products were used. All monetary values were corrected to December 2023, using the Broad National Consumer Price Index (IPCA) – the official inflation index in Brazil. The corrected values were converted to dollars (Central Bank) on 29 December 2023 (1.00 USD = 4.84 BRL).

The calculation of the expenditure on insecticides for corn leafhopper control (GICP) in the state of Paraná was performed based on Eqn (1):

(1) $$\text{GICP}=\sum_{i=1}^{n}\sum_{j=1}^{n}\sum_{k=1}^{n}\frac{{\left(\mathrm{GI}\right)}_{\mathrm{ijk}}}{a_{i}}$$

where i represents the municipalities; j represents the months of the agricultural year; k represents the insecticides used to control corn leafhoppers; GI represents the expenditure on insecticides only, obtained by multiplying the price of the input by the quantity of the input applied; 𝑎 represents the area planted with corn in each municipality.

The calculation of expenditure on corn leafhopper control per municipality (GCC) was performed using Eqn (2):

(2) $$\mathrm{GCC}=\sum_{j=1}^{n}\sum_{k=1}^{n}\frac{{\left(\mathrm{GI}\right)}_{\mathrm{jk}}}{a}$$

where j represents the months of the agricultural year; k represents the insecticides used to control corn leafhoppers in the municipality; GI represents the expenditure on insecticides, obtained by multiplying the price of the input by the quantity of the input applied in the municipality; 𝑎 represents the area planted with corn in the municipality. To facilitate the interpretation of GCC data and patterns, these were represented in the form of maps for each agricultural year.

To analyze whether there is any spatial pattern in the expenditure on leafhopper control in the state of Paraná, Exploratory Spatial Data Analysis (ESDA) was applied. This is considered a collection of techniques to describe and visualize possible spatial patterns. For its application, a first‐order queen‐type contiguity matrix was used. In this matrix, only municipalities that have a direct physical border with a given municipality analyzed were classified as being neighbors.

The first technique used to identify a spatial pattern in the GCC was the spatial autocorrelation coefficient known as Global Moran's I,^12^ calculated according to Eqn (3):

(3) $$I=\frac{n\sum_{i=1}^{n}\sum_{j=1}^{n}w_{\mathrm{ij}}\left(y_{i}-\bar{y}\right)\left(y_{j}-\bar{y}\right)}{\left(\sum_{i=1}^{n}\sum_{j=1}^{n}w_{\mathrm{ij}}\right)\sum_{i=1}^{n}{\left(y_{i}-\bar{y}\right)}^{2}}$$

where n represents the number of municipalities in the state of Paraná; $w_{\mathrm{ij}}$ assumes a weight equal to one for municipalities when municipality j is a neighbor, according to the first‐order queen‐type matrix, of the municipality i analyzed, and zero otherwise; $y_{i}$ represents the GCC of the municipality i; $y_{j}$ represents the GCC of the neighboring municipality j; $\bar{y}$ represents the average GCC of the state of Paraná with the control of the corn leafhopper. The statistical significance of the Global Moran's I coefficient is an indication of the existence of a spatial pattern of the GCC in the state.

Subsequently, Moran's I was decomposed to identify local patterns of spatial homogeneity or heterogeneity, the so‐called Local Moran's I. At this stage, the Local Indicator of Spatial Association – LISA^13^ was applied, enabling obtention of statistically significant patterns, such as High‐High (HH), Low‐Low (LL), which denote spatial clusters, and High‐Low (HL) and Low‐High (LH), which indicate spatial outliers. To calculate the Global Moran's I and Local Moran's I, a significance level of 5% was considered to confirm or reject the existence of spatial patterns in the state of Paraná.

## 3 RESULTS

### 3.1 Planted area, production and productivity of corn

**Figure 2:**
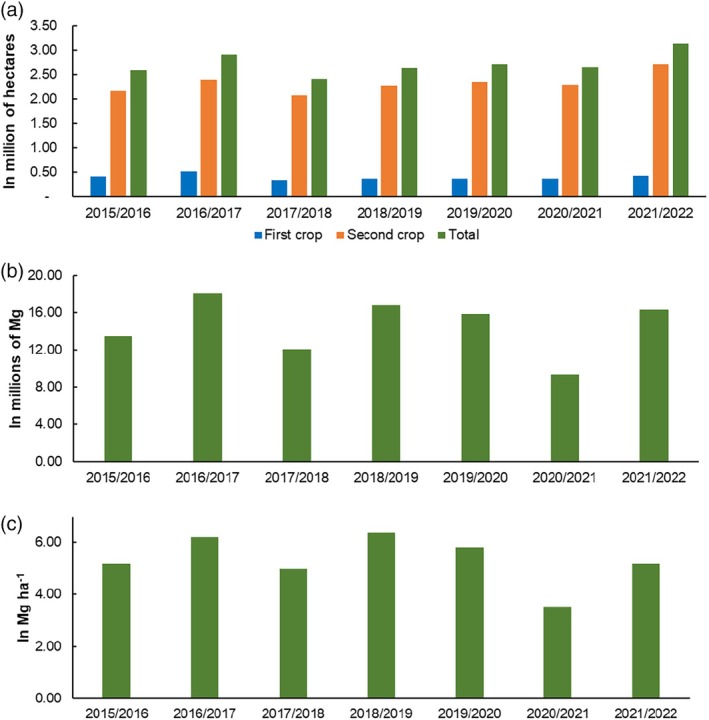
(a) Planted area, (b) production and (c) productivity of corn in the state of Paraná from the 2015–2016 to 2021–2022 crop years. *Source*: Based on data from the Secretariat of Agriculture and Supply of the state of Paraná.^11^

The area planted with corn in Paraná underwent some changes between the agricultural years 2015–2016 and 2021–2022 (Fig. 2(a)). It is possible to observe that, between the agricultural years 2015–2016 and 2021–2022, there was an increase in the total area (sum of the areas of the first crop and the second crop) planted with corn of approximately 17.5%, going from 2.59 million hectares to 3.14 million hectares. The smallest area planted with corn was recorded in the 2017–2018 agricultural year, when it was less than 2.41 million hectares. Furthermore, of the total area allocated to corn cultivation, around 85% was cultivated in the second crop.

Corn production in Paraná showed fluctuations during the analyzed period (Fig. 2(b)), with production below 15 million Mg in the years 2015–2016 and 2016–2017 and 9.32 million Mg in 2020–2021. Productivity also showed fluctuations during the analyzed period, the highest productivity was recorded in the year 2018–2019, with 6.39 Mg ha^−1^, and the lowest in the year 2020–2021, with 3.51 Mg ha^−1^ (Fig. 2(c)).

**Figure 3:**
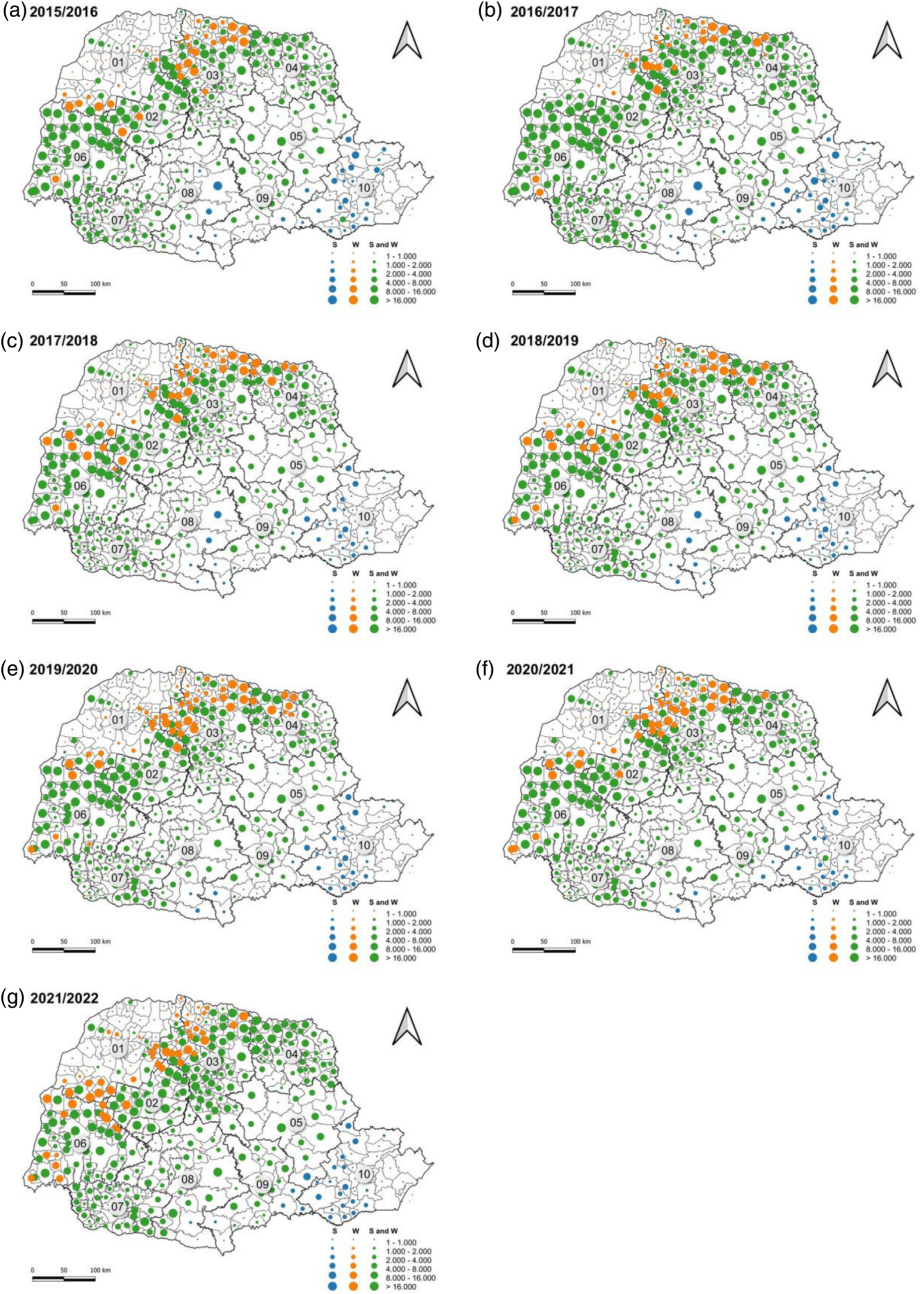
Distribution of the area planted with corn in hectares in the state of Paraná from the 2015–2016 to 2021–2022 crop years. *Source*: Based on data from Secretariat of Agriculture Supply of the state of Paraná.^11^ S represents corn only in the first crop; W represents corn only in the second crop; 01, northwest Paraná; 02, central‐west Paraná; 03, north central Paraná; 04, Pioneer north Paraná; 05, central‐east Paraná; 06, west Paraná; 07, southwest Paraná; 08, central‐south Paraná; 09, southeast Paraná; 10, Metropolitan of Curitiba.

Between the agricultural years of 2015–2016 and 2021–2022, there were no major changes in the spatial dynamics of the area planted with corn in Paraná (Fig. 3). Most municipalities produced corn in both the first and second crops, with the exception of some municipalities in the southeast and east regions of Paraná, which produced corn only in the first crop, and some municipalities in the north and west regions that produced corn only in the second crop. Our data also indicate that, over the period under analysis, the municipalities with the largest corn production areas, exceeding 16 thousand hectares, are located in the west and north regions of Paraná (Fig. 3). The municipalities with the smallest cultivation areas, less than 8 thousand hectares, are located in the northwest, south, center‐south, and southeast regions of Paraná.

### 3.2 Expenditure on insecticides for corn leafhopper control

**Figure 4:**
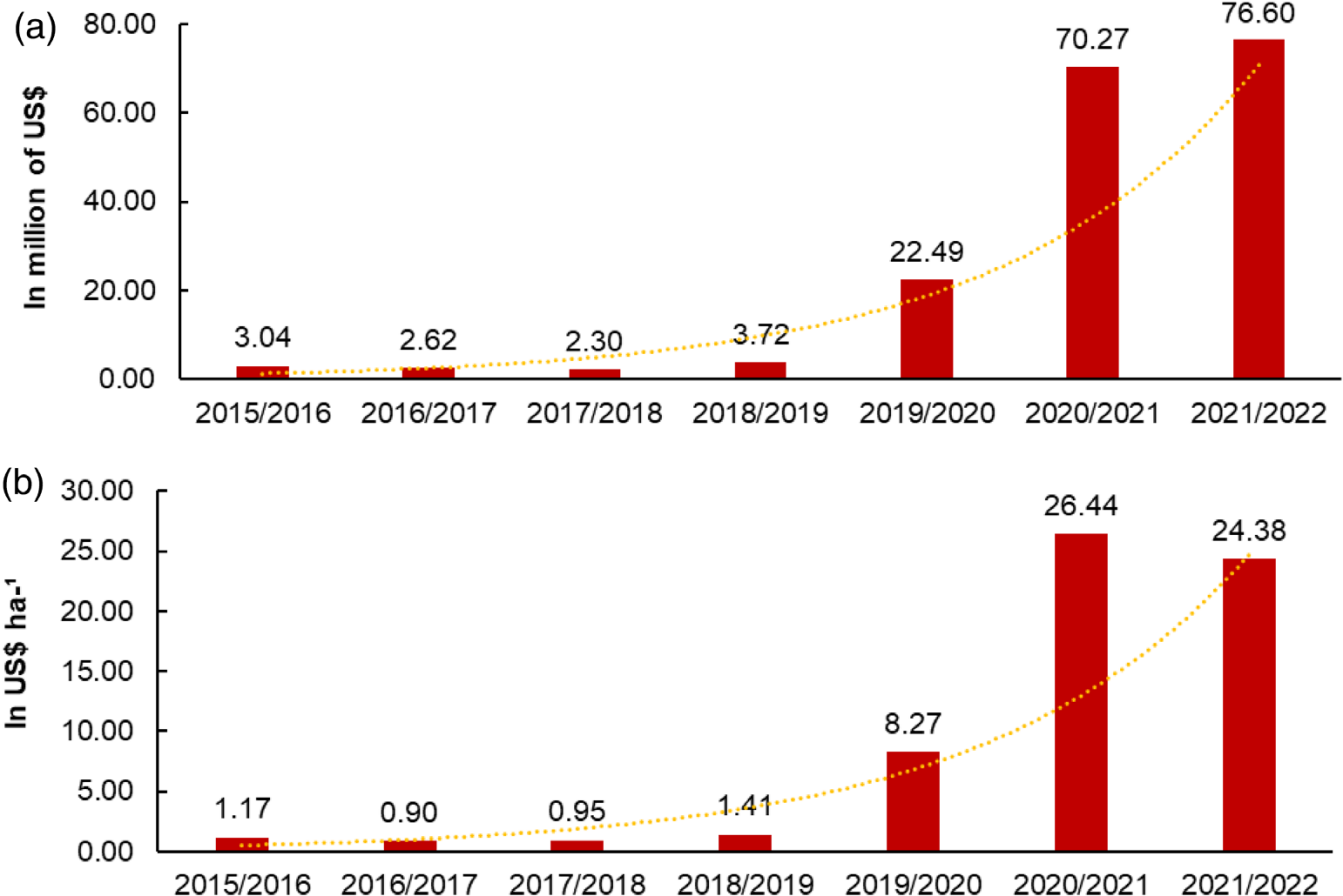
(a) Total and (b) per hectare expenditure on insecticides to control corn leafhoppers in the state of Paraná, from the 2015–2016 to 2021–2022 crop years. *Source*: Based on data from the Paraná Agribusiness Defense Agency.

Between the 2015–2016 and 2018–2019 agricultural years, the GICP was less than US$ 4 million in the state of Paraná (Fig. 4(a)). In 2019–2020, the GICP rose to US$ 22.49 million, representing an increase of 462.25% compared to the previous agricultural year. In 2020–2021 and 2021–2022, the GICP exceeded US$ 70 million, representing an increase of 212.45% when compared to the 2019–2020 agricultural year. The increase in the GICP from 2015–2016 to 2021–2022 was 2419%.

When analyzing the expenditure on insecticides for corn leafhopper control per hectare (GICH), increases from US$ 1.41 ha^−1^ in 2018–2019 to US$ 8.27 ha^−1^ in 2019–2020 (Fig. 4(b)) were observed, representing an increase of 487%. In the 2020–2021 agricultural year, the GICH reached US$ 26.44 ha^−1^, an increase of 219.71% compared to the previous year. In the 2021–2022 agricultural year, the GICH was US$ 24.38 ha^−1^, showing a reduction of 3.78% compared to the 2020–2021 agricultural year. In general, it can be seen that between the agricultural years 2015–2016 and 2021–2022 there was an increase in GICH of 1984%.

### 3.3 Active ingredients used to control corn leafhoppers

Between the agricultural years 2015–2016 and 2021–2022, the number of products used to control corn leafhoppers in Brazil increased by up to three times, with the number of chemical compositions increasing from five in 2015–2016 to 15 in 2021–2022 (Table 1). However, even with the increase in the number of chemical compositions recommended for corn leafhopper management, a large part of the GCC in Paraná was concentrated in a few compositions, suggesting a preference for the use of certain products, despite the greater variety of options available for pest control.

**Table 1:** Expenditure on insecticides for corn leafhopper control by commercial products in the state of Paraná from the 2015–2016 to 2021–2022 crop years, in US$

Active ingredient	2015–2016	2016–2017	2017–2018	2018–2019	2019–2020	2020–2021	2021–2022
Acephate 250 g kg^−1^ + Bifenthrin 250 g kg^−1^	X	X	5637.87	470529.47	2892109.23	13892788.63	10266931.99
Acephate 750 g kg^−1^	X	X	X	1361.09	4451609.07	9090188.91	3871664.40
Acephate 850 g kg^−1^ + Bifenthrin 30 g kg^−1^	877506.30	748111.94	X	X	X	X	X
Acephate 970 g kg^−1^	X	X	X	1679.00	5611832.16	13151517.72	21763833.86
Acetamiprid 75 g L^−1^ + Fenpropathrin 112,5 g L^−1^	X	X	X	X	175187.88	1764032.62	1496233.55
*Beauveria bassiana*	X	X	850.68	877.60	30228.59	2268420.70	3301992.07
Bifenthrin 50 g L^−1^ + Imidacloprid 250 g L^−1^	2176987.09	1877112.02	1660595.57	2124364.18	5992315.03	17441091.10	11587747.36
Bifenthrin 50 g L^−1^ + Carbosulfan 150 g L^−1^	X	X	4976.69	312728.94	1059370.95	5783255.36	8497069.49
Cypermethrin 40 g L^−1^ + Profenophos 400 g L^−1^	X	X	X	X	111665.80	432037.73	954578.81
Soybean oil methyl ester	249.08	X	X	X	X	321.39	X
Ethiprole 200 g L^−1^	X	X	X	X	X	496068.06	2431721.63
Imidacloprid 100 g L^−1^ + beta‐Cyfluthrin 12,5 g L^−1^	X	X	624382.05	808089.78	2166512.81	5691268.05	5376560.32
*Isaria fumosorosea*	X	X	X	X	X	261400.13	503427.06
Lambda‐Cyhalothrin 48 g g L^−1^ + Dinotefuran 84 g L^−1^	X	X	472.85	1173.58	6802.55	11109.86	749582.81
Methomyl 215 g L^−1^	X	X	X	X	X	X	5549949.99
Nonyl phenoxy poly ethanol	41.11	201.31	X	X	X	X	X
Mineral oil	96.70	X	X	X	X	X	X
Profenophos 500 g L^−1^ + Lufenurom 50 g L^−1^	X	X	X	X	X	X	1725.32
Thiamethoxam 500 g L^−1^	X	X	X	X	X	X	245658.88
Total expenditure	3054880.28	2625425.27	2296915.72	3720803.65	22497634.08	70283500.26	76598677.55

*Source*: Based on data from the Paraná Agribusiness Defense Agency.

*Note*: X indicates that the product was not recommended for the given agricultural year.

The chemical composition of Bifenthrin 50 g L^−1^ + Imidacloprid 250 g L^−1^ was predominant in the GCC of Paraná between the agricultural years of 2015–2016 and 2018–2019, corresponding to 72% of the GCC in the first 3 years and 57.1% in 2018–2019. From 2019–2020 onwards, other compositions began to be used, such as Acephate 970 g kg^−1^ and Acephate 250 g kg^−1^ + Bifenthrin 250 g kg^−1^, at the same time that Bifenthrin 50 g L^−1^ + Imidacloprid 250 g L^−1^ fell to around 25% of the GCC in this period, and together these compositions came to represent more than 50% of the GCC.

### 3.4 Spatial distribution of expenditure on insecticides for corn leafhopper control

In the analysis of GCC per hectare, it can be observed that, in the first agricultural years, a large part of the municipalities presented values between US$ 0 and US$ 10.00 ha^−1^. However, over the years, the percentage of municipalities in these categories decreased, while the percentage of municipalities in the categories with higher GCC values increased (Table 2). In the agricultural years 2015–2016 to 2018–2019, on average, approximately 31% of the municipalities in Paraná did not register GCC. However, in the agricultural year 2019–2020, approximately 15% of the municipalities did not present GCC, and in the agricultural year 2020–2021 this percentage fell to 8%. In the final year analyzed, 2021–2022, only 4% of the municipalities in Paraná did not register GCC.

**Table 2:** Number of municipalities in Paraná according to the spending ranges for leafhopper control per hectare from the 2015–2016 to 2020–2021 crop years

US$ ha^−1^	2015–2016	2016–2017	2017–2018	2018–2019	2019–2020	2020–2021	2021–2022
0	151	121	124	113	57	31	14
0.01–10.00	241	273	273	278	232	71	87
10.01–20.00	6	2	1	7	75	84	116
20.01–30.00	1	1	0	1	16	70	72
30.01–60.00	0	1	1	0	13	93	56
60.01–120.00	0	1	0	0	5	38	34
> 120	0	0	0	0	1	12	20

*Source*: Based on data from the Paraná Agricultural Defense Agency.

In the agricultural years 2015–2016 to 2018–2019, 68% of municipalities presented GCC values between US$ 0.01 ha^−1^ and US$ 30.00 ha^−1^ and less than 1% of municipalities presented GCC values above US$ 30.00 ha^−1^. In the 2019–2020 agricultural year, approximately 81% of municipalities presented GCC values between US$ 0.01 ha^−1^ and US$ 30.00 ha^−1^, approximately 3.26% of municipalities presented GCC values between US$ 30.01 ha^−1^ and US$ 60.00 ha^−1^, and 1.5% presented GCC values above US$ 60.01 ha^−1^. In the 2020–2021 agricultural year, approximately 56% of municipalities presented GCC values between US$ 0.01 ha^−1^ and US$ 30.00 ha^−1^, around 23% of municipalities presented GCC values between US$ 30.01 ha^−1^ and US$ 60.00 ha^−1^, and 13% presented GCC values above US$ 60.01 ha^−1^. In the 2021–2022 agricultural year, approximately 69% of municipalities presented GCC values between US$ 0.01 ha^−1^ and US$ 30.00 ha^−1^, approximately 14% of municipalities presented GCC values between US$ 30.01 ha^−1^ and US$ 60.00 ha^−1^, and 14% presented GCC values above US$ 60.01 ha^−1^.

**Figure 5:**
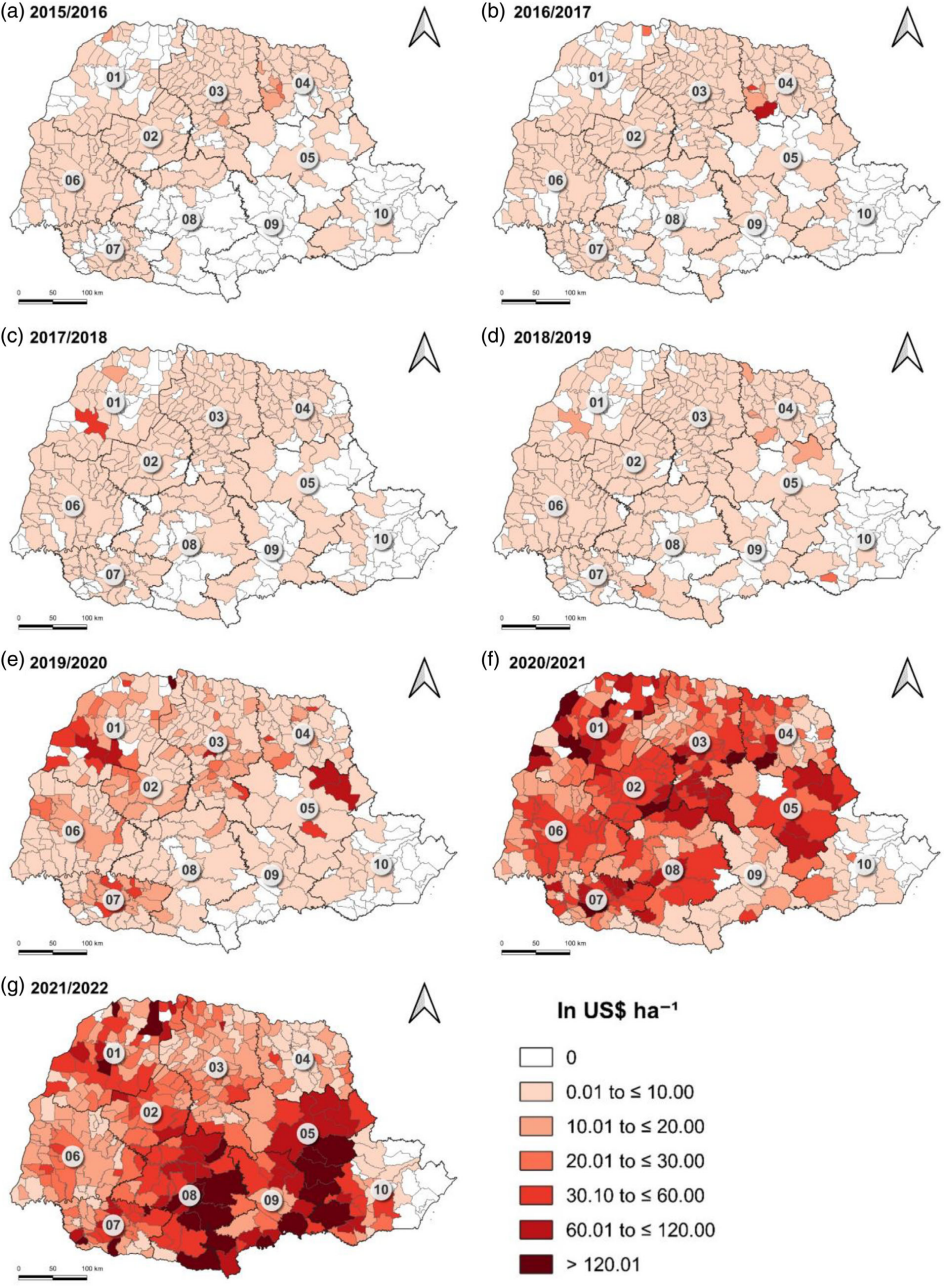
Expenditure per hectare on corn leafhopper control in the state of Paraná from the 2015–2016 to 2021–2022 crop years. 01, northwest Paraná; 02, central‐west Paraná; 03, north central Paraná; 04, Pioneer north Paraná; 05, central‐east Paraná; 06, west Paraná; 07, southwest Paraná; 08, central‐south Paraná; 09, southeast Paraná; 10, Metropolitan of Curitiba.

In the 2015–2016 agricultural year, it was observed that the municipalities that presented a GCC were concentrated in the west and north regions of Paraná (Fig. 5(a)). In the 2016–2017 agricultural year, a concentration of municipalities with a GCC was noted in the west, north, and south regions of Paraná (Fig. 5(b)). Furthermore, the two municipalities that presented the highest GCC values in Paraná are located in the north: Sapopema (US$ 80.75 ha^−1^) and Santa Cecília do Pavão (US$ 44.35 ha^−1^). In the 2017–2018 agricultural year, the GCC distribution remained similar to that of the previous year, however the municipality of Umuarama presented a GCC value of US$ 32.03 ha^−1^ (Fig. 5(c)).

In the 2018–2019 agricultural year, a GCC was observed in almost all municipalities in Paraná (Fig. 5(d)), however, there were no municipalities with a GCC value above US$ 30.00 ha^−1^. In the 2019–2020 agricultural year, approximately 86% of the municipalities in the state of Paraná with corn cultivation recorded a GCC value. The municipalities with the highest GCC values, above US$ 60.01 ha^−1^, were concentrated in the west, north, and center regions of the state (Fig. 5(e)), among them, Marumbi (US$ 87.60 ha^−1^), Umuarama (US$ 79.41 ha^−1^), and Inajá (US$ 121.52 ha^−1^).

In the 2020–2021 agricultural year, 92% of the municipalities in Paraná presented a GCC value. The municipalities with the highest GCC values, above US$ 120.01 ha^−1^, were concentrated in the northwest, center, and southwest regions of the State (Fig. 5(f)). The highest GCC values were presented in the municipalities of Mauá da Serra (US$ 186.81 ha^−1^), Sapopema (US$ 186.31 ha^−1^), and Saudade do Iguaçu (US$ 200.99 ha^−1^). However, in this same agricultural year, a lower concentration of municipalities with a GCC above US$ 60.00 ha^−1^ was observed in the west and southeast regions of Paraná.

In the 2021–2022 agricultural year, 96% of the municipalities presented a GCC value. However, unlike previous years, the municipalities with the highest GCC values, that is, above US$ 120.01 ha^−1^, were more concentrated in the center‐south and east regions of Paraná (Fig. 5(g)). Among these municipalities, the following stand out: Carambeí (US$ 201.25 ha^−1^), Castro (US$ 238.93 ha^−1^); Reserva do Iguaçu (US$ 202.96 ha^−1^); and Guarapuava (US$ 218.09 ha^−1^).

**Figure 6:**
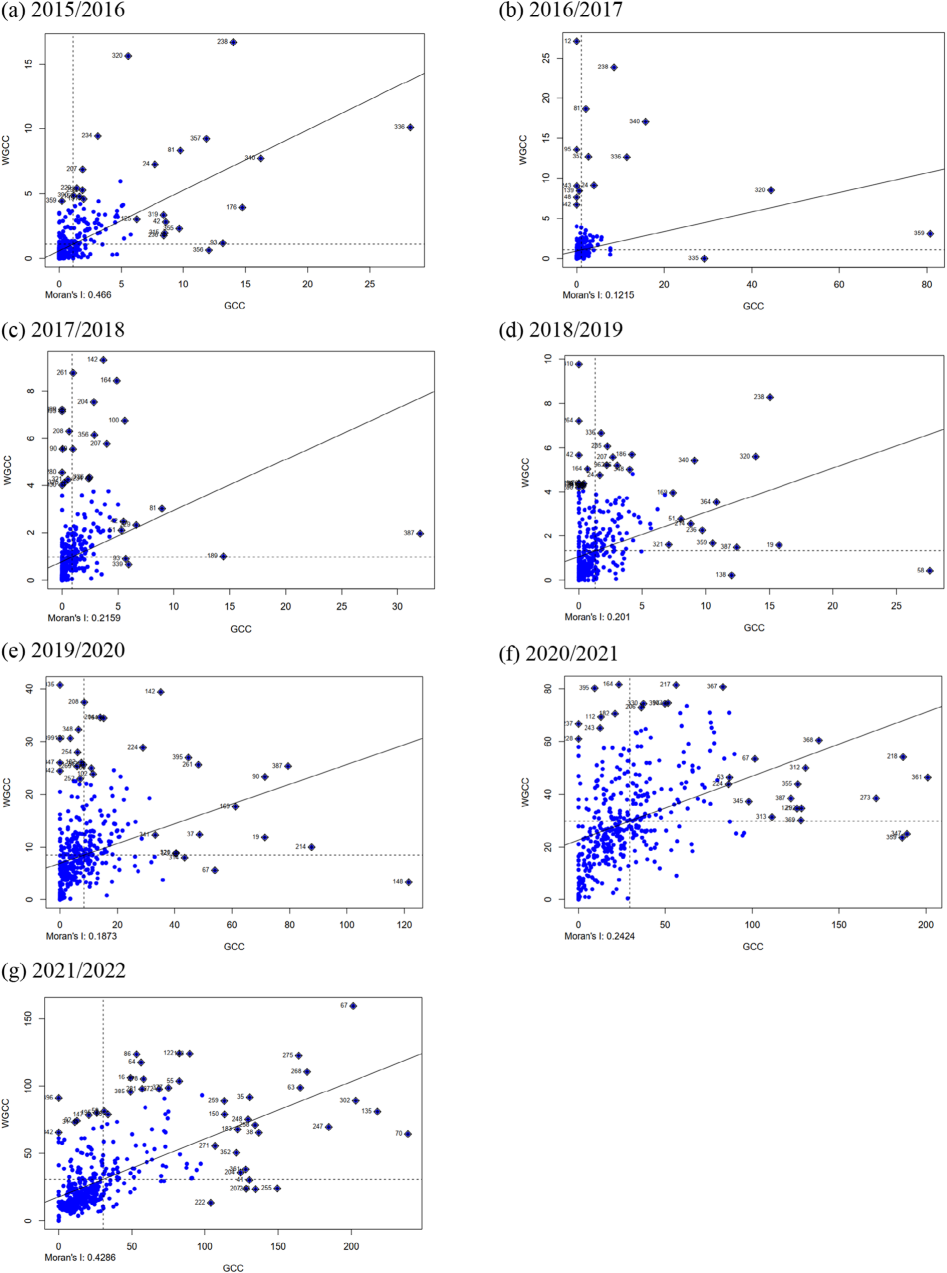
Moran's I for spending on corn leafhopper control in the Paraná from the 2015–2016 to 2020–2021 crop years. All Moran's I values were statistically significant at 5%. The GCC axis represents the expenditure on corn leafhopper control in the municipality in relation to its WGCC neighbors, according to the first‐order queen matrix.

The ESDA demonstrated the existence of positive spatial autocorrelation (> 0) in the GCC of Paraná (Fig. 6). The estimated Moran's I coefficients were positive and statistically significant at the 5% level. The values obtained for Global Moran's I for each agricultural year were I=0.46 in the agricultural year 2015–2016 (Fig. 6(a)), *I* = 0.12 in 2016–2017 (Fig. 6(b)), *I* = 0.22 in 2017–2018 (Fig. 6(c)), *I* = 0.20 in 2018–2019 (Fig. 6(d)), *I* = 0.19 in 2019–2020 (Fig. 6(e)), *I* = 0.24 in 2020–2021 (Fig. 6(f)), and *I* = 0.43 in 2021–2022 (Fig. 6(g)), indicating an increase in the correlation for the formation of HH or LL clusters.

**Figure 7:**
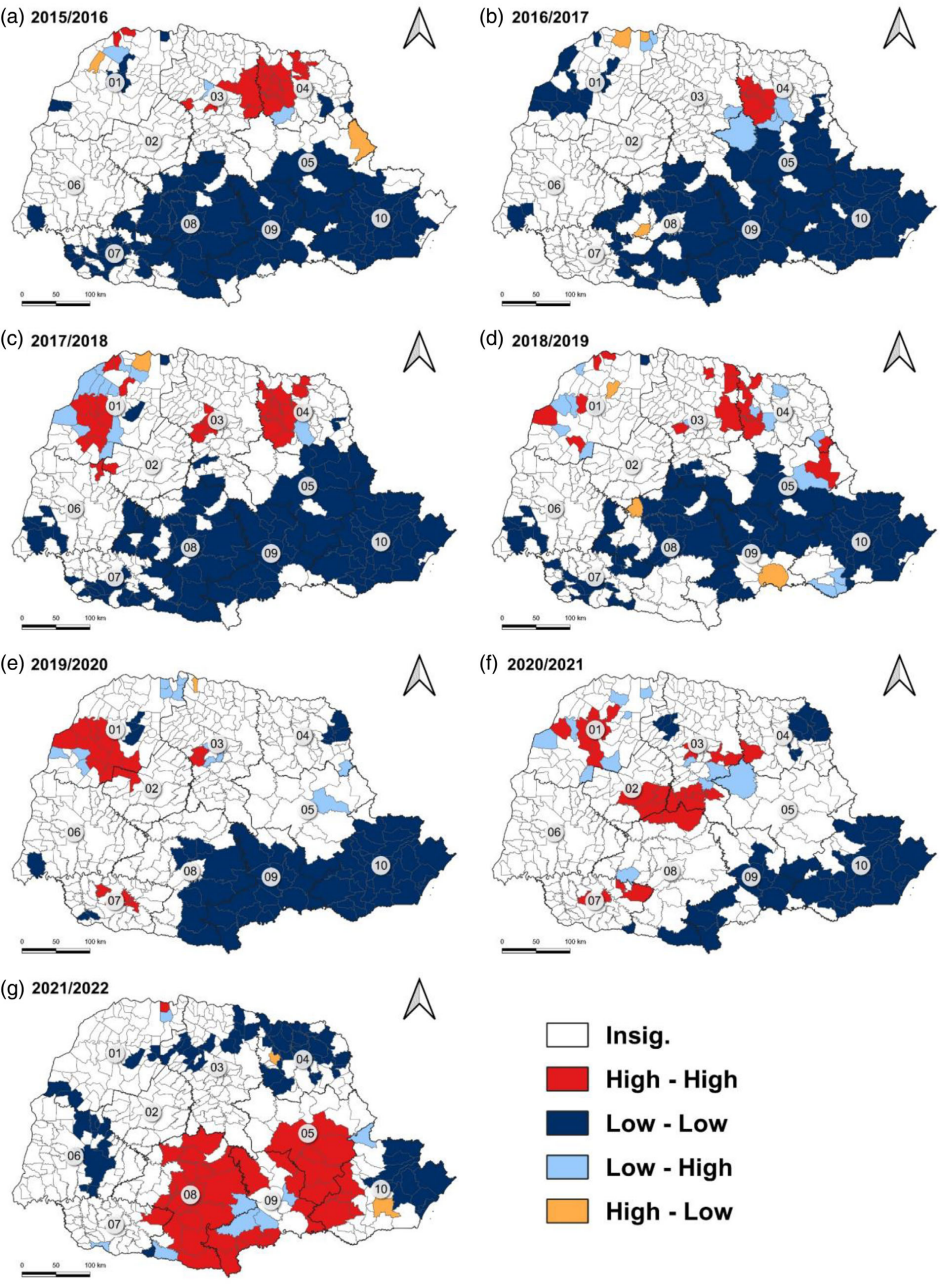
Local Indicator of Spatial Association (LISA) for expenditure on leafhopper control in the state of Paraná from the 2015–2016 to 2021–2022 crop years. High‐high indicates spatial clusters of high values, low‐low spatial clusters of low values, high‐low indicates that a spatial unit with a high value is surrounded by units with low values, and a low‐high spatial unit with a low value is surrounded by units with high values.

The spatial analysis of the GCC, using the LISA method, showed the formation of distinct clusters between the regions of the state (Fig. 7). In the first two agricultural years evaluated, 2015–2016 and 2016–2017, the formation of an HH cluster was observed predominantly in the north‐eastern region of the state, indicating that the municipalities in this region have significantly higher GCC values than the state average. In contrast, the formation of a small LL cluster in the northwest and a large LL cluster in the central‐south and eastern regions of Paraná was observed, showing that the municipalities in these regions present statistically lower GCC values than the state average (Fig. 7(a),(b)).

In the agricultural years of 2017–2018 and 2018–2019, the formation of HH clusters was observed in the northwest region of the state, where in previous years there were LL clusters. In addition, the intensification of the HH cluster was observed in the northeast region of Paraná. In this same period, it was possible to observe that the LL cluster in the southernmost regions of the state presented a smaller area, in relation to previous years (Fig. 7(c),(d)).

In the 2019–2020 agricultural year, a concentration of the HH cluster was observed in the northwest of the state. The HH clusters located in the northeast disappeared, while small HH clusters emerged in the southwest region of Paraná. The LL cluster present in the regions to the south of the state had a smaller area, being more concentrated in the southeast of the state (Fig. 7(e)).

In the 2020–2021 agricultural year, in addition to the presence of the HH cluster in the northwest and southwest of Paraná, a new HH cluster was formed in the central region of the state. The LL cluster in the southeast region presented further reductions in area, assuming the configuration of a narrow strip further to the south and southeast. In addition, the formation of an LL cluster was identified in the northeast region of the state (Fig. 7(f)).

In the 2021–2022 agricultural year, there was an inversion in the cluster patterns presented in the first years evaluated, with the formation of two large HH clusters in the south and southeast regions of the state. Due to the formation of these clusters, the LL cluster that previously occupied this area became concentrated in a small cluster further east of the state. Furthermore, the formation of LL clusters in the northern, north‐eastern, and western regions of Paraná was observed (Fig. 7(g)).

## 4 DISCUSSION

During the first four agricultural years analyzed, from 2015–2016 to 2018–2019, the GCC values remained relatively low and stable, and were concentrated in municipalities in traditional regions of intensive grain cultivation, that is, the north and west of Paraná – where corn is grown in both the first and second crops. This spatial pattern suggests that the occurrence of the leafhopper, in this period, was linked to the ‘green bridge’, which, according to Oliveira and Frizzas^4^ is a continuity in the food supply, provided by the uninterrupted cultivation of corn. The constant presence of the host in the field creates a favorable environment for the continuous reproduction of the pest, which contributes to the need to control leafhopper populations throughout the crops.

From the 2019–2020 agricultural year onwards, a significant increase in GCC values was observed, with expansion in different regions of Paraná. This increase intensified even further in the years 2020–2021 and 2021–2022. Some local factors may have contributed to this increase, such as the long period of drought and high temperatures recorded between 2019 and 2022. High temperatures directly influence insect biology, favoring faster insect reproduction, and allowing multiple generations to occur in a single crop.^14^, ^15^ As a consequence, there is more intense dissemination of pathogens related to the corn stunt complex in corn crops.^16^

**Figure 8:**
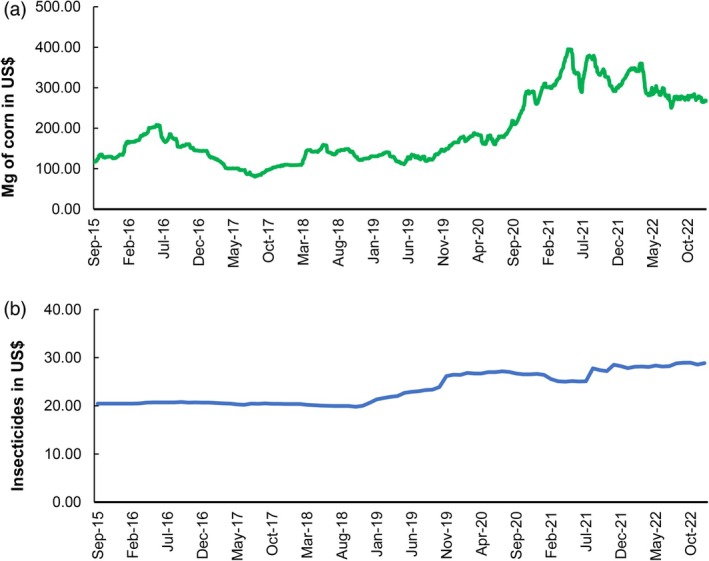
(a) Daily selling price of corn in megagrams (Mg) of grains, and (b) average price of insecticides^a^ in the markets of Paraná, from September 2015 to December 2022. *Source*: Based on price reports from the Paraná Agriculture and Supply Secretariat.^11^ ^a^Based on the average prices of the following insecticides: Acephate 250 g kg^−1^ + Bifenthrin 250 g kg^−1^, Acephate 750 g kg^−1^, Acephate 850 g kg^−1^ + Bifenthrin 30 g kg^−1^, Acephate 970 g kg^−1^, Acetamiprid 75 g L^−1^ + Fenpropathrin 112.5 g L^−1^, *Beauveria bassiana*, Bifenthrin 50 g L^−1^ + Imidacloprid 250 g L^−1^, Bifenthrin 50 g L^−1^ + Carbosulfan 150 g L^−1^, Cypermethrin 40 g L^−1^ + Profenophos 400 g L^−1^, soybean oil methyl ester, Ethiprole 200 g L^−1^, Imidacloprid 100 g L^−1^ + beta‐Cyfluthrin 12.5 g/L, *Isaria fumosorosea*, lambda‐Cyhalothrin 48 g L^−1^ + Dinotefuran 84 g L^−1^, Methomyl 215 g L^−1^, nonyl phenoxy poly ethanol, mineral oil, Profenophos 500 g L^−1^ + Lufenurom 50 g L^−1^, Thiamethoxam 500 g L^−1^.

Due to the long period of drought, it is possible to observe a reduction in corn production in 2020–2021 (Fig. 2(b)), which resulted in a low supply of grain to the market and an increase in its selling price. The price of corn grain reached up to US$ 400.00 per Mg in 2020–2021, while in previous years it did not exceed US$ 200.00 per Mg (Fig. 8(a)). The increase in grain prices may encourage farmers to expand cultivation areas, including less traditional regions for the crop, with a 16% increase in the area planted with corn being observed from the 2020–2021 agricultural year to the 2021–2022 agricultural year (Fig. 2(a)). However, the greater availability of hosts, due to the expansion of the planted area, may have contributed to the intensification of pest pressure in the field, increasing the demand for insecticides to control the leafhopper and to maintain high levels of productivity.

Another point observed is that a large part of the municipalities that presented their highest GCC values in recent years had corn cultivation areas of less than 16 thousand hectares, indicating that these municipalities are not traditional grain cultivation area and were influenced by the high price of grain in the period. As well as by the 40.85% increase in the average prices of insecticides between the 2015–2016 and 2021–2022 crop seasons (Fig. 8(b)). The municipalities with the highest GCC values belong to regions such as the northwest of Paraná, which is characterized by livestock farming and permanent crops, and the Metropolitan Region of Curitiba, characterized by family farming and forest areas. In recent years, these areas have shown the highest GCC values compared to the west region, which is characterized by intensive grain production and the use of high technology.^17^, ^18^ Thus, the high GCC values in these regions may be related to the lack of access to production technologies, such as cultivars resistant to the corn stunt complex, in addition to insufficient technical assistance and inadequate management of the corn leafhopper.

The increased use of insecticides also results in an increase in grain production costs, due to higher spending on inputs and agricultural operations. This has a direct impact on farmers' profits and the sustainability of corn cultivation. According to the study in a Londrina, Paraná, by Volsi,^19^ the first crop of corn had an average profitability of US$ 1740.42 ha^−1^ and an average production cost of US$ 1000.99 ha^−1^, resulting in a profit of US$ 739.43 ha^−1^ (values adjusted for December 2023). The second crop of corn had an average revenue of US$ 632.04 ha^−1^ and an average production cost of US$ 874.48 ha^−1^, resulting in a loss of US$ 242.44 ha^−1^.

Based on the current study, in municipalities with GCC values greater than US$ 120.00 ha^−1^, the cost of corn production could exceed US$ 1120.99 ha^−1^ in the first crop and US$ 994.48 ha^−1^ in the second crop. The first crop would have a reduction of more than 16% in profit and in the second crop the loss would increase by more than 50%. Furthermore, other studies have also shown that second crop of corn cultivation is high risk and tends to be loss‐making; however, this loss is diluted by the profit from other crops grown in the first crop.^20^ In this case, the increase in GCC not only impacts the profit from the corn crop itself, but also the profit of the entire production system.

The increase in GCC values also assumes that chemical control of corn leafhoppers may not be efficient under field conditions.^21^ The insecticide resistance is a factor contributing to the increasing problems with *D. maidis* and may lead to an increased use of insecticides to control this pest.^22^ This is attributed to several biological characteristics of the insect, such as its small size, migratory behavior, high reproductive capacity, and oviposition characteristics.^23^ Therefore, corn growers facing economic losses due to corn stunt disease should put into practice a six‐step management program to reduce insect vector populations and prevent pathogen spread, simultaneously adopting strategies such as removal of volunteer corn plants, reduction of planting period, use of tolerant corn hybrids, crop rotation with non‐grass species and protection of corn seeds and seedlings with chemical and/or fungal insecticides.^24^

Currently, there are 55 products registered for foliar spraying to control corn leafhoppers in Brazil.^25^ However, the current study identified the use of only 19 distinct chemical compositions, with three of these products accounting for more than 50% of the state's GCC. This suggests a concentration in the use of certain insecticides, possibly due to their perceived efficacy, or to factors such as cost and availability. However, prolonged use of the same mechanism of action can lead to the selection of resistant biotypes.^26^ This issue is worrying because similar cases have already occurred with other crop pests, such as the fall armyworm,^27^ making pest control in the field even more difficult.

In addition to the selection of resistant biotypes, the use of insecticides can cause environmental impacts when used indiscriminately, such as the reduction in natural enemies and pollinating insects, and toxicity to other animals.^28^ The use of biological insecticides could be an alternative to chemical insecticides and has been used in the field since the 2017–2018 agricultural year. However, its adoption remains insignificant when compared to the use of chemicals (Table 2), mainly due to its low efficiency in the field and the long insect mortality time. Under controlled conditions, the *Beauveria bassiana* isolate demonstrated approximately 50% efficacy in controlling the corn leafhopper, with an average insect mortality time of 9 days.^29^

Given the lack of studies on the control efficiency of chemical and biological insecticides, and also of economic studies on the damage caused by the stunting complex and insect control, it is important to recommend the use of integrated pest management (IPM) techniques, such as: the use of resistant cultivars, monitoring of the insect population, elimination of volunteer corn, and the rotation of active ingredients,^16^ in order to minimize dependence on the use of insecticides, reducing the risks and costs of corn production, and thus contributing to a more ecologically sustainable agriculture.

## 5 CONCLUSION

The current study analyzed expenditures on insecticides to control corn leafhoppers in Paraná in the agricultural years 2015–2016 to 2021–2022. The results indicate an increase of approximately 2419% in GCC values in this period, reflecting intensification of the use of insecticides.

The spatial distribution of expenditures indicates that the GCC has expanded beyond traditional growing regions, such as the west and north, concentrating high values in areas with a lower production history, such as the northwest and center‐south, possibly due to lower access to technical assistance and IPM technologies.

Furthermore, even with the increase in the number of products released for the control of leafhoppers over the years, spending has been concentrated on just three chemical compositions, which favors the selection of resistant biotypes and may make it difficult to control the pest in the future.

In view of these findings, technical assistance and the adoption of preventive measures, such as the use of tolerant cultivars, IPM practices, and diversification of active ingredients are essential to reduce dependence on insecticides, minimize environmental impacts, and ensure the economic sustainability of corn cultivation in the state.

## CONFLICT OF INTEREST

The authors declare no conflict of interest.

## Data Availability

The data that support the findings of this study are available from the corresponding author upon reasonable request.
